# Curriculum interventional cardiology—Austria

**DOI:** 10.1007/s00508-024-02475-6

**Published:** 2024-12-16

**Authors:** A. Kammerlander, R. Berger, R. K. Binder, J. Dörler, M. Frick, T. Gremmel, A. Mader, J. Kammler, A. Rab, A. Geppert, A. Schober, A. Niessner

**Affiliations:** 1 Division of Cardiology, Medical University of Vienna, Vienna, Austria; 2 Department of Internal Medicine 1, Hospital of Saint John of God, Eisenstadt, Austria; 3 Department of Cardiology and Intensive Care, University Teaching Hospital Klinikum Wels-Grieskrichen, Wels, Austria; 4 Department of Internal Medicine and Cardiology, Klinikum Klagenfurt am Wörthersee, Klagenfurt, Austria; 5 Department of Internal Medicine I and Cardiology, Academic Teaching Hospital Feldkirch, Feldkirch, Austria; 6 Department of Internal Medicine I, Cardiology and Intensive Care Medicine, Landesklinikum Mistelbach-Gänserndorf, Mistelbach, Austria; 7 Karl Landsteiner Society, Institute of Cardiovascular Pharmacotherapy and Interventional Cardiology, St. Pölten, Austria; 8 Karl Landsteiner University of Health Sciences, Krems, Austria; 9 Department of Cardiology and Internal Intensive Medicine, Kepler University Hospital, Medical Faculty, Johannes Kepler University, Linz, Austria; 10 Department of Internal Medicine I, Kardinal Schwarzenberg Klinikum, Schwarzach im Pongau, Austria; 11 3rd Department of Medicine, Cardiology with Intensive Care Medicine, Clinic Ottakring, Montleartstraße 37, 1160 Vienna, Austria; 12 Department of Cardiology, Klinik Floridsdorf, Vienna, Austria; 13 2nd Department of Medicine with Cardiology and Intensive Care Medicine, Vienna Healthcare Group Clinic Landstraße, Vienna, Austria

**Keywords:** Interventional cardiology, Training, on-duty shift skills, Interventional procedures, competence levels

## Abstract

The curriculum for interventional cardiology outlines a structured training program for advanced training in interventional procedures. It specifies requirements for candidates, trainers, and centers. The curriculum specifically defines learning objectives, competence levels, and essential skills needed for on-duty shift in the catheterization laboratory. The program is based on the European Core Curriculum, tailored to Austrian healthcare needs, and aims to ensure high-quality care.

**Supplementary Information:**

The online version of this article (10.1007/s00508-024-02475-6) contains supplementary material, which is available to authorized users.

## Content

1: Introduction and learning objectives
1.1: Rationale for an Austrian core curriculum for interventional cardiology
1.2: General aspects
1.3: Learning objectives
1.4: Categories and levels of competence
2: Knowledge in interventional cardiology
2.1: Anatomy and physiology of the cardiovascular system
2.2: Diagnostic modalities
2.3: Pharmacology
2.4: Coronary artery disease
2.5: Structural heart disease
2.6: Peripheral arterial disease
2.7: Complications of percutaneous cardiovascular interventions
2.8: Radiation safety
2.9: Quality assurance and research
3: Skills and on-duty shift skills
3.1: Essential skills with focus on on-call services
3.2: Extended skills
4: Behaviors and attitude
5: Requirements for candidates
6: Educational framework
7: Requirements for a training center
7.1: Interventional program
7.2: Equipment
7.3: Supervision
8: References.

## 1 Introduction and learning objectives

### 1.1 Rationale for an Austrian core curriculum for interventional cardiology

The field of interventional cardiology is rapidly evolving and requires an advanced set of knowledge and skills. The present Austrian core curriculum of interventional cardiology provides standards for training in interventional cardiology. It is based on the core curriculum for percutaneous cardiovascular interventions of the European Association of Percutaneous Cardiovascular Interventions (EAPCI) [1] and adapted to fit local requirements of the Austrian healthcare system. Other documents relevant for this curriculum include the European Society of Cardiology (ESC) core curriculum for the general cardiologist [2] and a statement endorsed by the American College of Cardiology and the American Heart Association on advanced training in interventional cardiology [3]. Also, the curricula for interventional training in cardiology by the German Society of Cardiology [4] and by the Swiss Society of Cardiology [5] were taken into account.

### 1.2 General aspects

Physicians licensed by the Austrian Medical Chamber are eligible to undergo training. The trainee must have the necessary level of linguistic skills in German to enable communication with patients and colleagues. Trainees should be exposed to various aspects of interventional cardiology, including inpatient/outpatient and emergency/elective care. These are listed in Table 1.

**Table 1:** Summary of the general aspects of training in interventional cardiology. Adapted from [1]

Area of training	General aspects of training
Continuous medical education	Structured learning, under supervision including explicit learning (journal club, postgraduate teaching, exercises in evidence-based medicine, discussion of guidelines for clinical practice, national/international symposia/congresses attendance) and implicit learning (ward rounds, case-based discussions, supervised acquisition of diagnostic and interventional skills)
Supervision and mentoring	Acute and elective cases with direct supervision, progressing from second to first and ultimately independent operator status
Research	Participation in clinical/translational research to enhance critical appraisal of evidence
Evaluation	Clearly defined for each individual, review/appraisal of their progress
Outpatient care	Preprocedural and postprocedural assessment
Acute coronary syndromes	Appropriate mix of inpatient/emergency and outpatient/elective care, including patients with acute coronary syndrome and out of hospital cardiac arrest
Percutaneous coronary interventions	Experience with different arterial access routes and exposure to several complex techniques
Structural interventions	Exposure to structural intervention is strongly recommended
Heart team	Regular participation in the heart team meetings

The training program should be clearly defined for each trainee, with regular review of their individual progress.

### 1.3 Learning objectives

Learning objectives represent specific aims the trainee has to reach by the end of the curriculum. These objectives are divided into three groups: 1) knowledge, 2) skills and 3) behaviors and attitudes.

Knowledge describes the requirements for trainees. The present curriculum defines the respective chapters for the intended learning goals. This knowledge includes disease pathobiology and concepts for treatment. It is important to highlight the need for long-term learning because even basic concepts of our understanding of a disease are subject to change based on emerging new evidence.

Skills represent the practical application of knowledge and are acquired from experience and training. These include the solution of practical challenges, clinical decision making, and performing specific procedures.

Behaviors and attitudes represent a commitment to perform excellent clinical care for the patient, taking ethical considerations and patient preferences into account.

### 1.4 Categories and levels of competence

It is important to highlight that first-hand exposure and practical experience are essential in learning special techniques. The number of cases performed by a trainee is certainly of importance but is not an exclusive measure of performance. Rather than a specific case volume needed to reach a certain competence level, such competence levels are defined for the individual progress of the trainee:

- Competence levels I and II
  The trainee must have acquired the experience in diagnosis and treatment for a referred patient.
  - LeveI I does not require any procedural skills, yet participation in related procedures during training may be valuable.
  - Level II of competency indicates acquisition of some procedural skills as operator, usually as assistant/second operator, obtained in the primary or external training centers.
- Competence level III
  The trainee has to be able to interpret clinical information, make treatment decisions, and perform the technique or procedure and manage related complications as first operator but still requiring direct supervision of a senior interventional cardiologist.
- Competence levels IV and V
  The trainee has to be able to interpret clinical information, make treatment decisions, and perform the technique or procedure and manage related complications as first operator, without direct supervision of a senior interventional cardiologist. Post hoc supervision including case review with senior colleagues is possible. Competence level V includes the ability to teach and supervise the technique or procedure to junior colleagues.

## 2 Knowledge in interventional cardiology

To obtain the highest benefit from the training to become an interventional cardiologist, a balanced relationship of responsibility and accountability is required between the trainee and the trainer. On the part of the trainee, a basic knowledge and understanding of physiology, anatomy, pathology, and pathophysiology must be assumed. Building upon these foundations, knowledge of interventional cardiology can be developed, allowing for the training and acquisition of skills. The details are described in the chapter parts I–V of the “2020 EAPCI core curriculum for percutaneous cardiovascular interventions” [1]. The EAPCI core curriculum provides a comprehensive overview of essential knowledge relevant to interventional cardiology.

In addition, to become an internationally recognized interventional cardiologist the European examination in core cardiology (EECC) is strongly recommended. The EECC includes basic knowledge in interventional cardiology. In addition, several ESC-organized courses as well as national programs approved by the working group for interventional cardiology (AGIK) will enhance the theoretical as well as practical knowledge in interventional cardiology.

The chapter parts I–V of the EAPCI core curriculum impart the following topics in brief:

### 2.1 Anatomy and physiology of the cardiovascular system

The anatomy and physiology of the cardiovascular system includes the structure and function of the heart, arteries, veins, microcirculation, valves and further, the pathophysiology of cardiovascular diseases such as atherosclerosis, hypertension, valvular dysfunction and heart failure. The ESC provides a wide range of information represented in the EECC curriculum. This knowledge can be obtained in different national courses.

### 2.2 Diagnostic modalities

Various diagnostic modalities are used in interventional cardiology, including electrocardiography (ECG), echocardiography, cardiac catheterization, angiography, intravascular ultrasound (IVUS), optical coherence tomography (OCT) and invasive physiological assessments (e.g., fractional flow reserve, FFR). Several national and ESC-organized courses impart this knowledge.

### 2.3 Pharmacology

Appropriate medication is indispensable for interventional cardiology. The safe use of antiplatelet agents, anticoagulants, vasodilators and vasoconstrictors is necessary and their potential complications must be known as well accurately managed, for example, bleeding and contrast-induced nephropathy.

### 2.4 Coronary artery disease

The diagnosis and management of coronary artery disease (CAD), including chronic coronary syndrome (CCS), acute coronary syndromes (ACS), and chronic total occlusions (CTOs) are cornerstones of interventional cardiology. This field contains the various treatment options for CAD, including percutaneous coronary intervention (PCI), coronary artery bypass grafting (CABG) and optimal medical therapy.

### 2.5 Structural heart disease

The diagnosis and management of structural heart disease, including valvular heart disease, congenital heart disease and left atrial appendage occlusion (LAAO), are of growing interest and new technologies are regularly implemented. For example, transcatheter aortic valve replacement (TAVR), mitral and tricuspid valve repair and replacement as well as closure of septal defects, have expanded the scope of interventional cardiologists.

### 2.6 Peripheral arterial disease

Also, part of the EAPCI curriculum is the diagnosis and management of peripheral arterial disease (PAD), including atherosclerosis, acute limb ischemia and chronic limb ischemia, including various treatment options for PAD, such as angioplasty, stenting and atherectomy.

### 2.7 Complications of percutaneous cardiovascular interventions

For a proper management of different complications of percutaneous cardiovascular interventions clinical knowledge and skills are needed. Additionally, a thorough reflection on any complications within the interventional team is crucial for effective management. Typical complications include bleeding, vascular access site complications, contrast-induced nephropathy and injury of coronary arteries.

### 2.8 Radiation safety

In addition to striving for maximum patient safety, due to the nature of working in a radiation area, personal safety is also extremely important. Therefore, the principles of radiation safety in interventional cardiology, including the risks associated with ionizing radiation, radiation protection measures and dose reduction techniques must be trained.

### 2.9 Quality assurance and research

The field of interventional cardiology is constantly evolving and to keep up with these changes it is necessary to rely on research, registries and clinical trials to evaluate the safety and efficacy of new techniques and devices.

## 3 Skills and on-duty shift skills

### 3.1 Essential skills with focus on on-call services

Diagnostic coronary angiography is the first clinical and practical milestone on the way to becoming an independent interventional cardiologist. Prerequisites for starting training in cardiac catheterization comprise profound knowledge and understanding of the human vasculature (in particular coronary anatomy), indications, contraindications, potential complications and pitfalls of the procedure. Moreover, the trainee should already be able to analyze and interpret angiograms of normal and anomalous coronary arteries as well as those of coronary artery bypass grafts. Fortunately, the complication rates of diagnostic coronary angiography are low; however, severe acute complications, such as anaphylaxis, vasospasm, embolization, hemodynamic compromise, arrhythmia and vessel damage can still occur. As hemodynamic measurements, which require wiring of a coronary artery, are nowadays an important and increasingly applied part of many diagnostic procedures, even coronary artery dissection and perforation can very rarely occur during noninterventional cardiac catheterization. Finally, while severe bleeding complications are almost nonexistent in cases of radial artery access, they can become a major issue when femoral artery access is needed, specifically in older and frail patients. Therefore, an operator who is only cleared for diagnostic coronary angiography always requires a readily available back-up by a senior cardiologist who is proficient in interventional cardiology and able to manage complex complications as well as iatrogenic consequences of cardiac catheterization.

Institutions with a 24‑h service for primary percutaneous coronary intervention (PPCI) require on-call staff who are proficient in invasive management of acute coronary syndromes including all severity grades, such as cardiogenic shock and cardiorespiratory arrest. Physicians who cover the on-call service need to be independent operators with extensive experience in PPCI and be able to tackle possible complications of myocardial infarction as well as iatrogenic consequences of cardiac interventions. Based on the EAPCI document [1] at the end of training the trainee should be able to interpret clinical information, make treatment decisions and perform the technique or procedure and manage related complications as the first operator, without direct supervision of a senior interventional cardiologist (competence level IV). The competence level IV listed in Table 2 according to the current recommendations of EAPCI should also be met when performing on-call services without the back-up of senior operators.

**Table 2:** Level of Competence IV skills required for performing on-call services

Peripheral venous access
Radial access
Femoral access < 10F
Closure devices < 9F
Pericardiocentesis
Right and left hemodynamic assessment
Coronary angiography
Ventricular angiography
PCI in simple lesions
PCI in STEMI
PCI in NSTE-ACS
PCI in multivessel disease
PCI in bypass grafts
PCI in bifurcation lesions
PCI in LM
Invasive physiology (FFR, iFR, RFR and others)
OCT/OFDI
IVUS

*PCI* percutaneous coronary intervention, *STEMI* ST-elevation myocardial infarction, *NST-ACS* Non-ST elevation acute coronary syndrome, *LM* left main, *FFR* fractional flow reserve, *iFR* instantaneous wave-free ratio, *RFR* relative flow reserve, *OCT/OFDI* optical coherence tomography/optical frequency domain imaging, *IVUS* intravascular ultrasound

In particular, the independent on-call operator should be ready to tackle the following challenges: acquiring peripheral arterial access from the radial or femoral artery can be challenging in patients who are restless or in shock. Intubation of the left or right coronary artery during cardiopulmonary resuscitation may require unusual C‑arm positions or projection angles. Acutely occluded coronary arteries in patients with ST-segment elevation myocardial infarction are mostly based on ruptured previously nonstenotic soft plaques and are usually easier to cross than severely calcified complex lesions in non-ST-segment elevation myocardial infarction or chronic total occlusions; however, bifurcation lesions and complex coronary anatomies including coronary artery by-pass graft interventions may be needed in acute settings. Hemodynamic compromise and arrhythmia can occur during PPCI and require a prompt response. Placement of a temporary pacing wire in the right ventricle from a femoral or jugular approach is an obligatory skill for on-call operators as well as subxyphoidal pericardiocentesis in cases of pericardial effusion or tamponade. The transition of an interventional cardiologist in training to an independent on-call operator may require a phase in which a senior interventional colleague is available as a secondary back-up if the primary physician encounters an overwhelming situation. If a situation cannot be handled by PPCI in a center without on-site cardiac surgery a formal cooperation with a tertiary center is mandatory.

Competence level V includes the ability to teach and supervise the technique or procedure to junior colleagues. While a structured academic program may not be feasible in all centers, teaching of junior colleagues including an adequate teaching environment is important for all centers involved in training of interventional cardiologists and is explained in more detail in “Chap. 7”.

In centers where hemodynamically unstable patients are treated or which serve as back-up for other centers, operators should be additionally able to perform more advanced procedures (competence level III for training according to current EAPCI recommendations) as listed in Table 3.

**Table 3:** Skills required for centers treating hemodynamically unstable patients or serving as back-up for other centers

Femoral access ≥ 10F
Closure devices ≥ 9F
Peripheral angiography
Use of percutaneous mechanical hemodynamic support

### 3.2 Extended skills

Supplemental table 1 lists extended skills (competence levels I and II) for procedures not performed in all centers. To obtain these skills, centers are encouraged to provide structured training opportunities, such as clinical rotation to other centers or training courses.

## 4 Behaviors and attitudes

In addition to knowledge and skills, behaviors and attitudes are an equally important goal of the training of interventional cardiologists and are an inherent part of optimal interventional performance. This learning aim of the curriculum includes a professional relationship with the patient, life-long learning, a professional conduct of the procedure, collaboration with other healthcare professionals and a systematic quality management. Table 4 provides detailed information about the different aspects of behaviors and attitudes [1, 6].

**Table 4:** Different aspects of behaviours and attitudes relevant for the interventional training

Professional relationship with the patient	Respect, consideration, and empathy for patients, families, and all members of the healthcare team
	Freedom from prejudice and bias concerning age, sex, gender, religion, skin color, mother tongue, citizenship, education, profession, social status, appearance, clothing, mental fitness, addictions and political attitudes of patients and their relatives
	Professional communication with the patient and provision of balanced, readily understood and appropriate information for shared decision making
	Timely explanation of the benefits and risks of a procedure as basis for an informed consent including the pros and cons of alternative treatment options and the (long-term) pharmacotherapy associated with the procedure
	Strict adherence to the principle of maintaining patient comfort before, during and after an interventional procedure
Life-long learning	The readiness of the interventional cardiologist to maintain current standards and utilize clinical practice guidelines
	To keep up to date with contemporary data and commitment to dedicate sufficient time to continuously move up the learning curve
Professional conduct of the procedure	Review medical records before starting any PCI that the diagnosis, indications, and informed consent as well as all other prerequisites, are satisfactory
	Appropriate acquisition and balanced interpretation of noninvasive and invasive data as basis for the decision and planning of an interventional procedure
	Understanding, evaluation, planning and discussing an individualized approach to PCI, according to the type of procedure and the identification of risk factors of the patient
	A systemic approach to provide a high-quality procedure in a safe manner for the patient and the catheter laboratory team
	Learning and maintaining the appropriate skills of coping with potential complications
	Recognition and managing the risk of radiation to patient and personnel
	Involvement in the postprocedural management and treatment of the patient
Collaboration with other healthcare professionals	Cooperation with the multidisciplinary catheter laboratory team including nurses, technicians and other medical professionals
	Collaboration with other healthcare professional, e.g., in the heart team meeting and with other specialties in the field of cardiology
Systematic quality management	Complete documentation and communication of results of diagnostic findings and management strategies to patients and collaborating healthcare professionals in a timely manner
	Solicit and incorporate systematic feedback from patients, colleagues and other healthcare team members to improve clinical performance
	Recognize research as a pivotal activity in professional practice and maintain a positive attitude towards it

*PCI* percutaneous coronary intervention

## 5 Requirements for candidates

Interventional cardiology is a subspeciality within the field of cardiology that covers all invasive procedures starting with diagnostic catheterization and ending up in complex percutaneous coronary and structural heart interventions in patients with low functional capacity and sometimes critically ill patients.

To guarantee a comprehensive patient care and an adequate use of interventional techniques, trainees in interventional cardiology must be in an advanced stage of cardiological training, typically in the last year. Based on the recommendation in the EAPCI core curriculum, international fellows who start an interventional training in Austria should have completed their training in general cardiology in their home country, whereas a minimum of 48 months of specific cardiology training is a basic requirement.

Furthermore, experience with critically ill patients for 6 or more months is strongly recommended, whereas at least a 3-month experience in the management of intensive care unit (ICU) and/or cardiac care unit (CCU) patients is a minimum requirement.

Consequently, interventional cardiologists should be able to weigh up all aspects of patient care, invasive as well as noninvasive and they should not exclusively focus on interventional procedures.

## 6 Educational framework

The recommended duration for interventional training is a minimum of 2 years. Parts of previous training in a catheter laboratory setting (such as angiography semester or similar) can be credited.

During the training, the applicant should have a strong focus on performing PCI. A personal training plan, tailored to the specific needs of the applicant and the center, should take personal factors, including parental leave or family medical leave, into account. During the training, applicants should be exposed to the entire spectrum of PCI, including treatment of patients presenting with acute coronary syndrome.

During the course of the curriculum, reaching the competence levels (“see Part 1”) is the primary goal. Although total case numbers performed by an individual applicant may not be used as a sole marker for the training process, a minimum number of the following is recommended: A) 50 PCI cases as second/assisting operator, B) 100 PCI cases as first operator, of which C) at least 1/3 are performed in patients with acute coronary syndrome (NSTE-ACS [Non-ST elevation acute coronary syndrome] and STE-ACS). In selected cases a reasonable deviation from this recommendation may be possible. A logbook should be used to facilitate documentation of the learning progress. The director of the training facility is responsible for overseeing the individual’s progress and confirmation of the successful completion of the training and successful completion of the aforementioned procedures.

## 7 Requirements for a training center

### 7.1 Interventional program

Quality management is crucial for an interventional training program. Monthly case discussions including morbidity and mortality conferences provide a structured feedback on the quality of interventions. Furthermore, a radiation safety program is required. Regular and standardized electronic data acquisition with an annual measure of procedural volume and performance measures is an additional important tool for the transparent monitoring of quality. These data should be the basis of an ongoing research program including participation in randomized controlled trials. Journal clubs should form a structured opportunity to critically appraise new evidence and current guidelines. Compliance with further local standards including minimum procedural volumes is a further prerequisite for a training center.

### 7.2 Equipment

Up to date equipment is required to ensure the interventional safety and to perform state of the art interventions including calcium-modifying methods, intravascular imaging and invasive physiological assessment. Further equipment including percutaneous mechanical circulatory support devices is encouraged for advanced centers [1]. These should also provide the infrastructure for structural procedures.

### 7.3 Supervision

The basis for a high-quality training in interventional cardiology is supervision and mentoring. The number of (full-time equivalent) trainees should not exceed the number of trainers. Every training center should have at least 2 experienced trainers with an experience of more than 5 years exclusively dedicated to interventional cardiology with proficiency in interventional skills mentioned in “Part 1”. There should be one dedicated educational mentor for each trainee being responsible for the implementation of the structured training for each individual trainee. This may include the keeping of a logbook, coordination of external rotations, attendance at courses and congresses and organizing structured learning. All interventional cardiologists at the center should support the interventional training as clinical trainers. Educational mentors should be supervised by the catheter laboratory director who is responsible for the achievement of learning objectives of each trainee. When the catheter laboratory director is the mentor another trainer should be involved in the training of the respective trainee.

## Supplementary Information

Supplemental Table 1
