# Supplementary material for: Curriculum interventional cardiology—Austria
Source: Wien Klin Wochenschr. 2024 Dec 16;136(Suppl 19):725–31. doi: 10.1007/s00508-024-02475-6 (PMC11649714; doi:10.1007/s00508-024-02475-6)
Supplement: Supplementary file 1 — Supplemental Table 1 [file 508_2024_2475_MOESM1_ESM.docx]

Supplemental Table 1

Level of competence II:

(Performance as second operator II and/or with direct, proactive
supervision)

| **Skills (EPAs)** |
| --- |
| PCI in CTO |
| PCI with rotablator/orbital artherectomy |
| Balloon aortic valvuloplasty |
| TAVR |
| Balloon mitral valvuloplasty |
| Left atrial appendage occlusion |
| Atrial septal defect closure or patent foramen ovale closure |
| Cardiac catheterisation in “Grown Up Congenital Heart” patients |
| Transseptal puncture |
| Interpretation of Multi-Slice CT for TAVR |
| Interpretation of transoesophageal echocardiography for mitral procedure |

Level of competence I:

(No performance, even with direct supervision. Observation is recommended)

| **Skills (EPAs)** |
| --- |
| Cerebral angiography |
| Endomyocardial biopsy |
| Transcatheter mitral valve repair |
| Transcatheter mitral valve implantation |
| Transcoronary ablation of septal hypertrophy |
| Transcatheter pulmonary valve interventions |
| Closure of ventricular septal defect |
| Percutaneous treatment of paravalvular leaks |
| Transcatheter myocardial stem cell therapy |
| Supra-aortic interventions including acute ischemic stroke treatment |
| Aortic disease interventions (Thoracic Endovascular Repair of Aorta/ Endovascular Repair of Aortic Aneurysm) |
| Infra-aortic arterial disease interventions |
| Renovascular interventions for arterial hypertension |
| Percutaneous direct treatment for Acute Pulmonary Embolism |
| Balloon pulmonary angioplasty for “Chronic Thrombo embolic Pulmonary Hypertension” |
